# Hidden in Plain Sight: Comprehensive Molecular Phylogeny of Keroplatidae and Lygistorrhinidae (Diptera) Reveals Parallel Evolution and Leads to a Revised Family Classification

**DOI:** 10.3390/insects11060348

**Published:** 2020-06-04

**Authors:** Michal Mantič, Tomáš Sikora, Nikola Burdíková, Vladimir Blagoderov, Jostein Kjærandsen, Olavi Kurina, Jan Ševčík

**Affiliations:** 1 Department of Biology and Ecology, Faculty of Science, University of Ostrava, Chittussiho 10, 71000 Ostrava, Czech Republic; michal.mantic@gmail.com (M.M.); sikothomas@gmail.com (T.S.); burdikova@seznam.cz (N.B.); 2 Department of Natural Sciences, National Museums Scotland, Chambers Street, Edinburgh EH1 1JF, UK; v.blagoderov@nms.ac.uk; 3 Department of Life Sciences, Natural History Museum, Cromwell Road, London SW7 5BD, UK; 4 Tromsø University Museum, UiT—The Arctic University of Norway, P.O. box 6050 Langnes, NO-9037 Tromsø, Norway; jostein.kjarandsen@uit.no; 5 Institute of Agricultural and Environmental Sciences, Estonian University of Life Sciences, Kreutzwaldi st 5D, 51006 Tartu, Estonia; olavi.kurina@emu.ee

**Keywords:** predaceous fungus gnats, phylogenetics, classification, Sciaroidea, Keroplatidae, Lygistorrhinidae

## Abstract

We provide the first molecular phylogeny of Keroplatidae and Lygistorrhinidae, families of fungus gnats (Diptera: Bibionomorpha: Sciaroidea). Phylogenies reconstructed by Maximum Likelihood and Bayesian methods, based on four nuclear and four mitochondrial gene markers (5106 base pairs) sequenced for 75 genera and 105 species, show Keroplatidae as monophyletic only with the family Lygistorrhinidae included, herewith treated as the subfamily Lygistorrhininae stat. nov. The subfamily Arachnocampinae is retained in the family, although lowering its overall support. An early branching clade, comprising species of *Platyura* Meigen, 1803 and *Paleoplatyura melanderi* Fisher, 1941, forms subfamily Platyurinae Loew, 1850 stat. nov. The subfamilies Sciarokeroplatinae and Macrocerinae grouped together with three genera considered here as Keroplatidae *incertae sedis*. Subfamily Lygistorrhininae forms a sister clade to subfamily Keroplatinae, both retained monophyletic with high support. The traditional division of the subfamily Keroplatinae into the tribes Orfeliini and Keroplatini appears as outdated, resting largely on adaptive characters prone to parallel evolution. We find support for an alternative tribe corresponding to the *Cloeophoromyia*–*Asindulum* genus group, but a tribal reclassification of the Keroplatinae is left for future studies. The genus *Heteropterna* Skuse, 1888 is considered as identical with *Ctenoceridion* Matile, 1972 syn. nov.

## 1. Introduction

The infraorder Bibionomorpha is one of the largest groups within the Diptera [1], currently including nine extant families plus several unplaced genera (Sciaroidea *incertae sedis*). With nearly 1000 species described in almost 100 genera, the family Keroplatidae is one of the most diverse families in this infraorder. New species and even genera within the family are continuously being described so the real number of species and genera is supposed to be much higher [2,3,4,5,6]. The family Keroplatidae is currently divided into four subfamilies—Arachnocampinae, Keroplatinae, Macrocerinae, and Sciarokeroplatinae [2,7,8].

**Figure 1:**
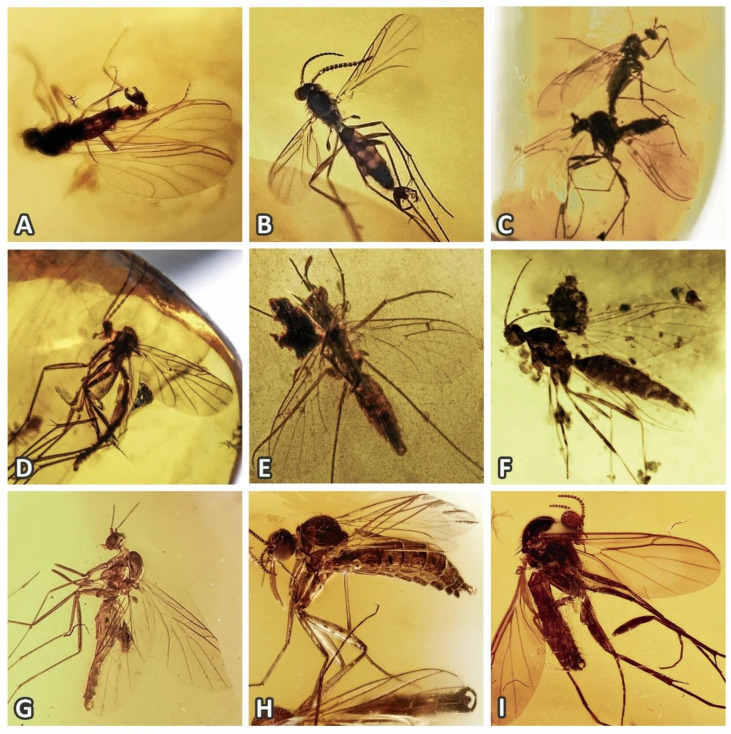
Examples of fossil Keroplatidae and related taxa (specimens from private collection of J. Ševčík): (**A**) Male of an undescribed Cretaceous genus of primitive Sciaroidea, representing one of possible ancestors of Keroplatidae (Burmese amber); (**B**) Male of an undescribed genus of Keroplatidae, possessing characters of both keroplatids and lygistorrhinids (Burmese amber); (**C**) Male and female of *Burmazelmira* sp., family Archizelmiridae (Burmese amber); (**D**) Male of *Palaeoplatyura* sp. (Burmese amber); (**E**) Male of an undescribed genus near *Platyura* (Burmese amber); (**F**) Example of a specimen possibly representing a female of *Burmacrocera petiolata* Cockerell, 1917 (Burmese amber); (**G**) Male of *Macrocera* sp. or a related genus (Burmese amber); (**H**) Male and female of *Paleognoriste* sp. (Baltic amber); (**I**) Male of a genus of Orfeliini (Baltic amber). All photos by J. Ševčík, except for (H) and (I) by M. Veta (www.ambertreasure4u.com).

The origin of this family dates back to the Early Cretaceous period (145–100 mya), with the oldest described species being *Hegalari minor* Blagoderov and Arillo, 2002; *H. antzinako* Blagoderov and Arillo, 2002 (both approx. 105.3–99.7 mya, found in Cretaceous Alava amber of Spain); *Schlueterimyia cenomanica* Matile, 1981 (approx. 99.7–94.3 mya, found in Cretaceous amber of France); and *Burmacrocera petiolata* Cockerell, 1917 (approx. 99.7–94.3 mya, found in Cretaceous Burmese amber in Myanmar). The first two of these fossil genera belong to the subfamily Macrocerinae, while the latter is currently placed in the tribe Orfeliini of Keroplatinae [2,3]. However, there is a large number of undescribed or unrecorded taxa of Keroplatidae and related taxa, especially in the mid-Cretaceous Burmese amber (see Figure 1), considered as essential for understanding the origins and diversification of recent families of Sciaroidea [9]. The Cretaceous family Archizelmiridae (see *Burmazelmira* sp. in Figure 1C as an example) also shows some affinities to the Keroplatidae and can potentially be considered as its ancestor or even a subgroup or extinct offshoot [10]. The oldest fossils placed into the family Lygistorrhinidae are from Lebanese Amber, ca. 130.0–125.5 mya [9].

Representatives of Keroplatidae can be found in all zoogeographical regions, with the highest number of species being described from the Palaearctic and Neotropical regions. The subfamily Arachnocampinae is known only from the Australasian region, with nine species known so far [11], and the subfamily Sciarokeroplatinae nowadays includes three species described from China, Japan and Taiwan [8,12]. The remaining subfamilies have a worldwide distribution.

**Figure 2:**
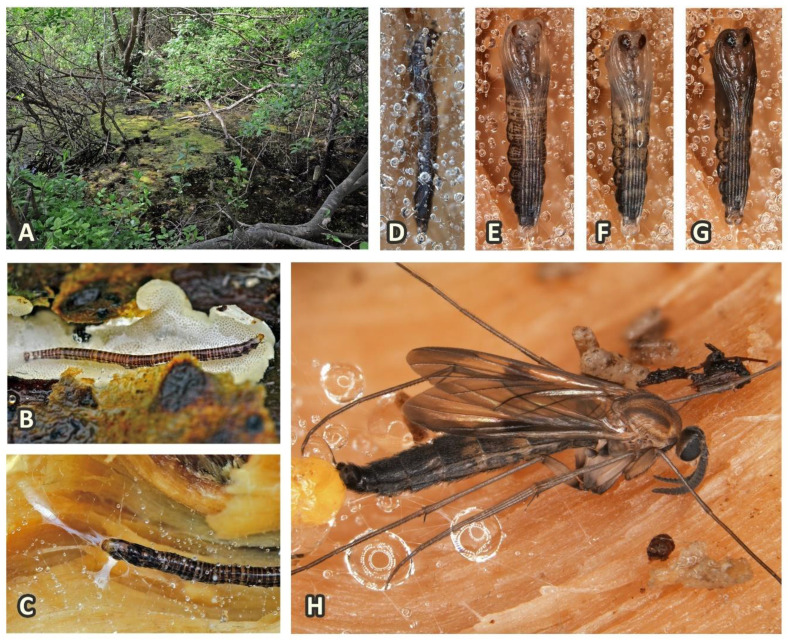
Biology and immature stages of Keroplatidae. This example follows a larva of *Cerotelion striatum* (Gmelin, 1790) through pupation to hatching. (**A**) Larval habitat in swampy shrubs of *Salix caprea* inside a limestone quarry in southern Sweden in early June 2012; (**B**) Larva feeding on an unidentified wood-growing fungus on a decaying *Salix caprea* branch partly submerged into water; (**C**) The larva net-spinning and moving around inside a web on the branch; (**D**) Pre-pupation took place under a dense web; (**E**–**G**) Daily photos of pupal maturing; (**H**) The adult male hatched only five days after pupation. All photos by J. Kjærandsen.

The biology of keroplatids remains mostly unstudied and the immature stages of the majority of its genera are completely unknown. While the trophic strategies of larvae appear to be relatively diverse, adult feeding is almost unrecorded, except for flower visiting in some genera [12,13,14]. Active pollination by sexual deception of the orchid genus *Pterostylis* is documented for *Xenoplatyura conformis* (Skuse, 1888) [15]. Predation is considered by Matile as the primary type of larval trophic strategy, while mycophagy or sporophagy emerged secondarily [16]. Only in some species the biology has been described in more detail. For example, the ability of bioluminescence and its genetic basis is well-studied in *Arachnocampa* Edwards, 1924, so-called glowworms [11,17], but also in several genera of Keroplatinae (e.g., [2,18]). In the subfamily Keroplatinae, larval association with wood-decaying fungi is known for several species (Figure 2), especially in the tribe Keroplatini, while representatives of the second tribe, Orfeliini, are considered primarily carnivorous [16]. Some adult keroplatids imitate wasps (e.g., *Nicholsonomyia vespiformis* Tonnoir, 1929), while other species have myrmecophilous or myrmecophagous larvae (e.g., *Proceroplatus* Edwards, 1925 and *Platyceridion* Tollet, 1955, cf. [19,20]). Some species of *Lyprauta* Edwards, 1931 and *Proceroplatus* have been found in plant greenhouses where they are likely to feed on insect pests [21]. Larval stages of the genus *Planarivora* Hickman, 1965 are known as endoparasites of terrestrial planarians [22]. The biology of the subfamily Sciarokeroplatinae remains still unknown, but the adults in Taiwan occur in enormous numbers in montane fir forests with old decaying trees, so the immature stages are possibly associated with dead wood and mycelia (Ševčík, personal observation). The family Lygistorrhinidae belongs to the few extant families of Diptera, still with completely unknown immature stages. The biology of the adults is also largely unrecorded, except for the observation of one species feeding on flowers [23].

The family Keroplatidae was initially classified as a subfamily in the family Mycetophilidae sensu lato [24], but it was subsequently raised to the family level, together with the other former subfamilies, now classified as families within the superfamily Sciaroidea [25,26,27]. Only few studies have been devoted to the classification and phylogeny of Keroplatidae, including their position within the Bibionomorpha, with one remarkable exception, the monograph by Matile [2]. This book covers all the higher taxa of Keroplatidae, except for the tribe Orfeliini.

As the closest relatives of Keroplatidae were mostly considered Diadocidiidae [2,16,28,29], Bolitophilidae [30] or Ditomyiidae [31]. These studies were based on morphological characters except for the molecular study by Wiegmann et al. [30]. In the latter study, the genus *Arachnocampa* was chosen as the only representative of the family Keroplatidae, so this could lead to the potentially misleading result (see the discussion in [1]). Several studies pointed to a possible close relationship of Keroplatidae with the family Lygistorrhinidae [1,25,32,33,34], based on both morphological and molecular data, although this view (presented by Tuomikoski [25]) was criticized by Thompson and Matile [2,35]. 

This study is an integral part of a long-term project aimed at molecular phylogenetic reconstruction of the infraorder Bibionomorpha and its particular families (see [1,36,37]). We present here the first comprehensive molecular phylogeny of the entire family Keroplatidae, based on a broad taxon and gene sampling. At the same time, several additional specific questions have arisen during this study, which need further separate studies, focused on particular subgroups of Keroplatidae. This paper, thus, can serve as a new starting point or work plan for future studies on this family. Where possible, molecular data are discussed and compared with morphological ones. This paper is, thus, a good example of when molecular data help to reconsider the diagnostic and systematic importance of some morphological characters.

We aimed to elucidate especially the following issues:

1. The monophyly and delimitation of the families Keroplatidae and Lygistorrhinidae and their position within the infraorder Bibionomorpha.
2. The monophyly, delimitation and interrelationships of the subfamilies and other higher taxa within Keroplatidae.
3. The phylogenetic position of various problematic genera of Keroplatidae (e.g., *Arachnocampa*, *Burmacrocera* Cockerell, 1917, *Chetoneura* Colless, 1962, *Kibaleana* Kurina, Mantič and Ševčík, 2017, *Paleoplatyura* Meunier, 1899, *Sciarokeroplatus* Papp and Ševčík, 2005, *Schizocyttara* Matile, 1974).
4. The inclusion of fossil taxa, either described or recently discovered undescribed ones, in the study of the phylogeny of Keroplatidae and Lygistorrhinidae.

## 2. Materials and Methods

A total of 105 species (Keroplatidae + Lygistorrhinidae + outgroup taxa) were used for the phylogenetic analysis (Table S1). The closely related families Bolitophilidae, Ditomyiidae, Diadocidiidae, Mycetophilidae, Sciaridae, Cecidomyiidae and Bibionidae were selected as outgroups, as well as several species from the Sciaroidea *incertae sedis* group. Most of the specimens used in this study were collected by Malaise traps during the years 2006–2019. All the specimens were identified by Michal Mantič and Jan Ševčík, and are stored in the comparative collection of the Ševčík Lab (University of Ostrava), if not stated otherwise. In some cases, we also obtained sequences from the GenBank database.

Most of the material used in this study was Ethanol stored (70% to 96%) or pinned dry. For DNA extraction, we used NucleoSpin Tissue Kit (Macherey-Nagel, Düren, Germany) following the manufacturer’s protocol. For polymerase chain reactions (PCR), we used ddH_2_O, buffer PCRBIO HS Taq Mix Red and pairs of primers for each of the eight gene markers (Table S2). When the required product was successfully amplified, we purified these regions with a Gel/PCR DNA Fragments Extraction Kit (Geneaid, New Taipei City, Taiwan) using the manufacturer’s protocol. Purified products were then sequenced by Macrogen Europe (Amsterdam, the Netherlands). Sequences were manually controlled using SEQUENCHER 5.0 (Gene Codes Corporation, Ann Arbor, MI, USA) or SeqTrace 0.9.0. (Guralnick Lab, Gainesville, FL, USA), respectively. Several sequences were taken from unpublished transcriptomic data prepared for a future study. For transcriptome sequencing, the specimen was put in RNAlater^®^ (Sigma-Aldrich, St. Louis, MO, United States) and then RNA was extracted using Trizol^®^ (Thermo Fisher Scientific, Waltham, MA, United States). Final sequences were deposited in GenBank, affiliated with accession numbers (Table S1). All the sequences were double checked using one gene trees, where possible errors could be seen.

Alignments of all genes were created using MAFFT version 7 [38] on the MAFFT server (http://mafft.cbrc.jp/alignment/server/) using L-INS-i algorithm, and then, manually edited. In the case of rRNA genes, alignments were subsequently submitted to the GBLOCKS program [39], available on the Gblocks server. We found out the less stringent conditions (allowed smaller blocks, allowed gap positions within the final blocks and allowed less strict flanking positions) outperformed the more stringent condition (do not allow many contiguous nonconserved positions) as well as not using GBLOCKS at all (data not shown). Protein-coding genes were checked based on amino acid translations in AliView and were not subsequently submitted to GBLOCKS.

To test whether our data were saturated, we conducted Xia′s nucleotide substitution saturation test in the software DAMBE 5.6.14 [40,41]. We separately analyzed rRNA genes and codon positions of protein coding genes using only the fully resolved sites and the empirical proportion of invariant sites estimated from the data. We found out that third positions of cyt b (cytochrome b) and MCS (molybdenum cofactor sulfurase) genes were substantially saturated, so these third positions were excluded from the subsequent analyses. The final data matrix thus consisted of 5106 characters: 12S (mitochondrial 12S ribosomal RNA)—345 bp, 16S (mitochondrial 16S ribosomal RNA)—327 bp, 18S (nuclear 18S ribosomal RNA)—1038 bp, 28S (nuclear 28S ribosomal RNA)—1322 bp, CAD (carbamoylphosphate synthetase-like protein)—642 bp, COI (cytochrome oxidase subunit I)—654 bp, cyt b (third positions removed)—284 bp, and MCS (third positions removed)—468 bp.

To select the best-fit model and partitioning scheme, we analyzed our data in PartitionFinder2 using the greedy search algorithm [42] and under the corrected Akaike information criterion. We did not include the option +G+I of rate heterogeneity because this approach has been demonstrated to result in undesirable interactions among parameters [43]. In the case of BI, we limited our search only to models which present in MrBayes. PartitionFinder recommended dividing our data by gene and by codon position (no merging of partitions at all). The best model selected was mostly GTR+G, with the exception of 28S, MCS_2 (TVM+G) and COI_3, cytb_2 (TRN+G) for ML analysis. For BI, only the GTR+G model was selected.

The dataset was analyzed using the Maximum Likelihood (ML) method and Bayesian Inference (BI). The ML method was conducted using IQ-TREE 2 [44], with partitions and models specified by PartitionFinder and with an edge-linked proportional partition model. Branch supports were evaluated using 1000 ultrafast bootstrap [45]. Additionally, preliminary analyses using single gene alignments were performed in IQ-TREE 2 to check for potential errors. MrBayes 3.2.7 [46], carried out on the CIPRES Science Gateway v.3.3 [47], was used for BI method. Two independent runs with four chains were run for 30 million generations using the MCMC method. The convergence of the runs was checked in Tracer 1.7 [48]. The first 30% were discarded as burn-in.

As a root, we used a member of the family Bibionidae, *Bibio marci* (Linnaeus, 1758), which is considered to be sister family to all the of Sciaroidea families (e.g., [1]). The node support values are given in the form: ultrafast bootstrap 2 (ufboot2)/posterior probability (PP).

Phylogenetic trees were visualized using Interactive Tree Of Life (iTOL) [49]. The tree presented is Maximum likelihood topology with node support values from both the BI and ML analyses. The ML and BI topologies obtained were similar, with only minor differences in several terminal branches. The BI tree is presented in supplementary Figure S1. The final tree was then manually designed in Adobe Photoshop CS6.

## 3. Results and Discussion

### 3.1. Delimitation and Monophyly of Keroplatidae

From the morphological point of view [2], the monophyly of Keroplatidae, in its currently accepted broader sense (i.e., including Arachnocampinae and Macrocerinae), is based on larval synapomorphies only, even though immature stages of the majority of extant genera of Keroplatidae and related taxa are still unknown, including the entire family Lygistorrhinidae, all the genera of the Sciaroidea *incertae sedis*, as well as all fossil taxa of Sciaroidea. While the external morphology of adult keroplatids is very diverse with respect to the form of antennae, mouth parts, wing shape and terminalia, their wing venation remains, with notable exceptions, surprisingly plesiomorphic and invariant throughout most of the family. This probably reflects fast evolution into a wider range of life mode adaptations compared with e.g., the largely fungivorous family Mycetophilidae, which complicates the discovery of clear-cut synapomorphies less prone to parallel evolution. With more and more genera having been described, a general picture gradually appeared where olfactory functions repeatedly were displaced from the mouthparts to the antennae, resulting in reduced palpi and pectinate antenna, a prolonged proboscis repeatedly evolved for flower feeding, and various other complimentary reductions probably occurring independently. Thus, it is likely that such functional characters have been overused as synapomorphies in the classical morphological phylogenies and classifications of the family.

This is exactly the situation where molecular data should be taken into consideration to carefully reconsider previous interpretations of morphological synapomorphies against new and independent evidence of monophyly based on DNA sequences.

**Figure 3:**
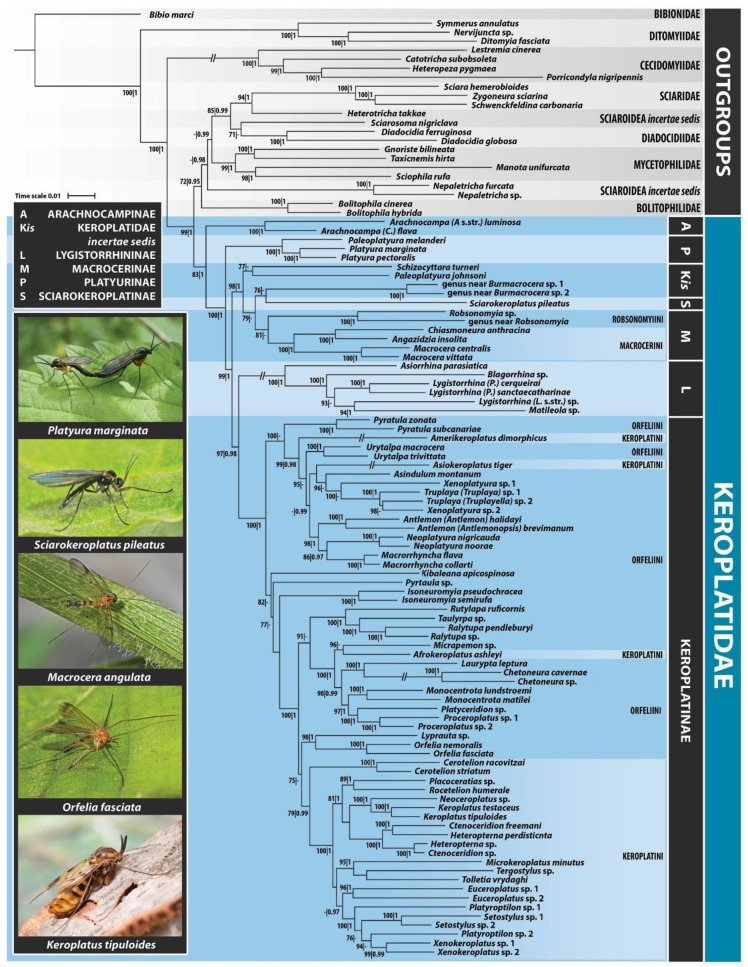
Maximum likelihood hypothesis for the family Keroplatidae based on the combined analysis of 105 taxa and 8 gene markers (12S, 16S, 18S, 28S, CAD, COI, cytb, MCS). Node support numbers refer to posterior probability (PP) over 0.95 and ultrafast bootstrap value (ufboot2) over 70.

Our analysis confirms the monophyly of the family Keroplatidae, including *Arachnocampa* (Figure 3), though with only moderate support (ufboot2 = 83/PP = 1). However, without *Arachnocampa*, the family is monophyletic with maximum support (ufboot2 = 100/PP = 1.00). This indicates that the position of *Arachnocampa* within the family Keroplatidae is not fully supported by current molecular data and requires further study. Based on these results, we propose as the best solution to maintain the subfamily status of Arachnocampinae within Keroplatidae, until more analyses are conducted, aimed specifically to elucidate the phylogenetic position of *Arachnocampa*.

Our analysis suggests that a sister group to Keroplatidae is not a single family but a clade containing all the other groups of Sciaroidea, except Ditomyiidae and Cecidomyiidae (Figure 3). Considering that *Arachnocampa luminosa* (Skuse, 1891) was initially placed in the genus *Bolitophila*, and the wing venation of both the genera show distinct similarities, the family Bolitophilidae is among the candidates to be expected as a possible sister group to *Arachnocampa* (see also [1,50]).

### 3.2. Subfamily Platyurinae Loew, 1850, Stat. Nov.

One of the most striking discoveries of the present study is the early branching of the well-supported clade (ufboot2 = 100/PP = 1), containing *Platyura* spp. and *Paleoplatyura melanderi* Fisher, 1941, from the rest of Keroplatidae (Figure 3).

The archaic origin of *Platyura* Meigen, 1803 (and related genera) is supported also by the fossil record (Figure 1), although most of these taxa still remain unnamed. While no species of true Keroplatinae is currently known from Cretaceous ambers, various representatives of genera related to *Platyura*, *Paleoplatyura* and *Macrocera* Meigen, 1803 are relatively frequent in Burmese amber (J. Ševčík, personal observation). This indicates that both the tribes of the subfamily Keroplatinae (Keroplatini and Orfeliini) represent a relatively young group, present only in the Tertiary (Baltic Amber) and later.

We take the opportunity here and establish a new subfamily of Keroplatidae, Platyurinae Loew, 1850 stat. nov., using the reinstated family group name introduced by Loew, 1850 (as Platyurina, hitherto a junior synonym of Orfeliini, see Sabrosky [51] (p. 252)) with the type genus *Platyura* (sensu authors, with type species *Platyura marginata* Meigen, 1803). Apart from DNA data, this subfamily can be defined as having at least the following combination of morphological characters (mostly plesiomorphic and none of them unique within Keroplatidae): basal section of wing vein media (M) present (at least as a weak and unsclerotized longitudinal fold), first radial cell present, broad semicircular (vein R_2+3_ oblique and ends in R_1_), veins M_1_, M_2_, M_4_, CuA and CuP all strong and reaching wing margin, antennae filiform, at least twice as long as the head but not longer than the body, male terminalia simple, *Macrocera*-type, with gonostylus apically bidentate.

An open question, beyond the scope of this paper, remains which other genera (both extant and fossil) should be assigned to the new subfamily. According to our results (Figure 3), *Paleoplatyura melanderi* and *P. johnsoni* Johannsen, 1910 are not closely related and they should not belong to the same genus. The type species of *Paleoplatyura* is a Baltic amber fossil, *P. macrocera* (Meunier, 1899), and its wing venation is more similar to the recent *P. johnsoni* than to *P. melanderi* (e.g., in vein R_2+3_ ending in C, not in R as in *P. melanderi*). *Paleoplatyura melanderi* thus should be either transferred to *Platyura* or to a new, very similar genus. This issue, however, needs to be clarified in a separate study, covering also *Paleoplatyura aldrichii* Johannsen, 1909 and two fossil species included in this genus.

### 3.3. Delimitation and Monophyly of Macrocerinae and Sciarokeroplatinae

According to Matile [2], the subfamily Macrocerinae is divided into two tribes, Macrocerini and Robsonomyiini. Some authors preferred to treat the subfamily Macrocerinae as a distinct family [52,53,54,55,56], but this view is no longer accepted. The vast majority of all described species in this subfamily are included in a rather heterogeneous genus *Macrocera*, where several morphologically distinct species groups would probably deserve at least subgenus rank [14]. Phylogenetic analyses of the Macrocerinae, based on morphological characters, were published by Matile [2] and for the tribe Robsonomyiini also by Ševčík [57].

The subfamily Sciarokeroplatinae until recently included only a single species, *Sciarokeroplatus pileatus* Papp and Ševčík, 2005, but two additional, morphologically very similar species were described by Saigusa [12]. As the subfamily may include (pseudo)cryptic diversity, a further molecular study is required to elucidate the species delimitation. Although Amorim and Rindal [28] and Amorim et al. [58] questioned the subfamily status of Sciarokeroplatinae, they neither presented any argument in support of their view nor alternative hypotheses.

The subfamily Macrocerinae, as currently understood [2], was found to be monophyletic in this study, but only with ML method and then, with low support (ufboot = 81, Figure 3). In the case of BI (Figure S1), this subfamily was found to be paraphyletic. However, the clade comprising also several other taxa (genus near *Burmacrocera*, *Paleoplatyura johnsoni*, *Sciarokeroplatus* and *Schizocyttara*) is strongly supported (ufboot2 = 98/PP = 1).

Our results thus suggest that the concept of the subfamily Macrocerinae should either be broadened appropriately (Macrocerinae sensu lato), to embrace the entire highly supported clade, or the concept of the subfamily can be retained, leaving the other genera within the broader clade unplaced to a subfamily, with the exception of *Sciarokeroplatus*, classified in the subfamily Sciarokeroplatinae. Trying to keep the number of changes in the current classification to a minimum and pending a separate study primarily focused on Macrocerinae and related taxa, we prefer to retain the current concept of Macrocerinae (sensu Matile [2], with the two tribes included) and not to create additional suprageneric taxa within this subfamily. Both the accepted tribes within the subfamily Macrocerinae sensu Matile [2] were found as monophyletic with high support (ufboot2 = 100/PP = 1, Figure 3).

The following taxa of Macrocerinae sensu lato do not form any monophyletic grouping in the present analysis (Figure 3), viz. *Sciarokeroplatus pileatus*, *Paleoplatyura johnsoni*, *Schizocyttara turneri* Matile, 1974, and a genus near *Burmacrocera*, although they constitute a broader clade together with Macrocerinae sensu Matile [2].

The overall habitus of *Sciarokeroplatus* is very different from all the other Keroplatidae, possessing several unique features within the family, like the absence of ocelli, short coxae, well-developed alula, and well-developed pulvilli in combination with large claws [8]. We thus consider as the best solution, for now, to leave this genus in its separate subfamily Sciarokeroplatinae, to emphasize its unusual combination of characters.

The position of *Paleoplatyura johnsoni* represents a real puzzle that cannot be resolved satisfactorily based either on current DNA data or morphology. The erection of the separate subfamily or tribe just for this species appears premature, so we currently prefer to leave it unclassified, as Keroplatidae *incertae sedis*. The same applies to *Schizocyttara turneri*. Both these genera were previously classified in the tribe Orfeliini (cf. [2,3]) and they share with Platyurinae and Macrocerinae (in part) the plesiomorphic character of having retained a distinct stem of M.

The taxonomy and phylogenetic position of the undescribed extant Oriental genus near *Burmacrocera*, including its delimitation against the fossil genus *Burmacrocera*, represents a rather complicated issue, requiring (among others) a re-description and clarification of the identity of *B. petiolata* Cockerell, 1917, a Burmese amber fossil, which is beyond the scope of this paper. A female possibly representing this species is depicted in Figure 1F.

Among other candidate genera to be included in this broader clade (Macrocerinae sensu lato), the South African genus *Asynaphleba* Matile, 1974 definitely deserves attention. This is a monotypic genus, currently placed in the tribe Orfeliini, based on a single species still known only from the male holotype, with remarkable wing venation (without radio-medial fusion but also without any trace of the basal part of the medial vein). However, without DNA sequences available for this species, we currently cannot decide unambiguously if this genus is related to Macrocerinae or not.

### 3.4. Subfamily Lygistorrhininae Edwards, 1925, Stat. Nov.

A total of six genera of Lygistorrhinidae were included in our dataset. All of them clearly form a monophyletic branch, with maximum support (ufboot2 = 100/PP = 1). This clade forms a sister group to the Keroplatinae clade, with high support (ufboot2 = 97/PP = 0.98). It is thus evident that this clade can no longer be considered as a separate family, at least until Arachnocampinae, Macrocerinae and other subfamilies are not considered as separate families too. Consequently, we propose the new status for this group, Lygistorrhininae stat. nov., as a subfamily of Keroplatidae.

This peculiar group of fungus gnats has been widely accepted as a separate family up to the present [59], although it was considered by some authors as part of the family Keroplatidae already in the second part of the 20th century [25,26]. Eight extant and eight fossil genera have been described within this group. The adults of lygistorrhinids are usually very rare (but some species may be locally common, see [35] p. 442), mostly tropical in distribution, and their immature stages and biology are completely unknown, which prevents including larval characters into the analysis.

As already mentioned above, the inclusion of Lygistorrhinidae into Keroplatidae or their close relationship is not a new idea. It was first proposed by Tuomikoski [25], and subsequently revived by Kallweit [34] and Ševčík et al. [1]. Bertone et al. [32] also showed in their molecular study that Lygistorrhinidae and Keroplatidae are closely related, although they did not discuss that issue in detail. Hippa [60] and Blagoderov et al. [61] studied the phylogeny and interrelationships of genera within Lygistorrhinidae, based on morphological characters, but they did not focus primarily on the phylogenetic placement of the entire group.

On the other hand, Matile [2,16], Hippa and Vilkamaa [29,31], and Amorim and Rindal [28] came to the alternative conclusion that Lygistorrhinidae forms a sister group to the family Mycetophilidae. Such a view is not supported by any molecular analysis (for a more detailed discussion on the topic, see Ševčík et al. [1]). Amorim and Rindal [28], for example, indicate the following two potential synapomorphies of their Mycetophiloidea (= Mycetophilidae + Lygistorrhinidae): narrow base (insertion) of the abdomen and very short male abdominal segment 8. Both of them are, however, present also in other groups of Sciaroidea, including Keroplatidae. Already, Tuomikoski [25] and Thompson [35] pointed out that the narrow abdominal insertion is found in the highly specialized families like Keroplatidae and Mycetophilidae. Amorim and Rindal [28] (p. 40) also refer (with reference to Matile [2], p. 376 and beyond) to extended clypeofrontal apodeme of larva as a synapomorphy uniting Mycetophilidae + Lygistorrhinidae, although the immature stages of Lygistorrhinidae are still unknown (see [2] p. 371).

We can find good support for the inclusion of Lygistorrhininae stat. nov. into Keroplatidae also in the fossil record. Several undescribed taxa of Keroplatidae (e.g., Figure 1B), relatively common in the mid-Cretaceous Burmese amber, show characters typical of both Keroplatinae and Lygistorrhininae, representing possible common ancestors of these taxa. The most striking is the presence of wing vein R_2+3_ and visible R-M fusion, typical of keroplatids, together with the rest of wing venation and terminalia typical of primitive lygistorrhinids, e.g., *Palaeognoriste* Meunier, 1904 (Figure 1B,H). Thus, it can be concluded that the reduced (invisible) central wing venation seen in extant genera is an apomorphic feature, most likely correlated to their small size and slender appearance, which cannot serve as a synapomorphy for the entire (family) clade as traditionally defined.

### 3.5. Monophyly and Interrelationships of Keroplatinae

Our results show that the traditional division of the subfamily Keroplatinae into the tribes Orfeliini and Keroplatini is outdated (Figure 3). While the tribe Keroplatini appears to be potentially monophyletic in a strict sense (after removing some problematic genera, like *Afrokeroplatus* Ševčík, Mantič and Blagoderov, 2015, *Amerikeroplatus* Fitzgerald, 2019 and *Asiokeroplatus* Ševčík, Mantič and Blagoderov, 2015), the tribe Orfeliini is clearly paraphyletic and does not deserve its tribal status, at least not until a substantial revision and redefinition is undertaken. A clade containing the two genera *Orfelia* Costa, 1857 and *Lyprauta*, is recovered as a sister group to Keroplatini, although with low support and only in the ML analysis (ufboot2 = 75). This entire latter clade appears further as a sister to the clade comprising many other Orfeliini genera, plus *Afrokeroplatus*. This entire assemblage of Keroplatini plus various Orfeliini genera is well supported (ufboot2 = 100/PP = 1), but we are currently unable to present any synapomorphy for this extended clade, pending revisionary work beyond the scope of this paper.

An alternative, relatively well-supported clade (ufboot2 = 99/PP = 0.98, Figure 3) of the Keroplatinae is worth attention, roughly corresponding to the *Cloeophoromyia*–*Asindulum* genus group of Matile [62], but including also the two recently described, monotypic genera *Amerikeroplatus* and *Asiokeroplatus*. Matile [2] (p. 169) listed two morphological synapomorphies for this clade; (1) the presence of an enlarged aedeagal complex, consisting of an elongated ejaculatory apodeme and an associated split furca (wrongly interpreted as gonocoxal apodemes in *Xenoplatyura* Malloch, 1928 by Blagoderov and Ševčík [63] —— Figure 85, protruding far inwards the abdomen, and (2) reduction of the gonocoxal apodemes. This group previously comprised 10 genera [62,64,65,66]: *Antlemon* Haliday in Loew, 1871, *Asindulum* Latreille, 1805, *Cloeophoromyia* Matile, 1970, *Macrorrhyncha* Winnertz, 1846, *Neoplatyura* Malloch, 1928, *Rhynchorfelia* Matile, 1988, *Rhynchoplatyura* Meijere, 1916, *Truplaya* Edwards, 1929, *Urytalpa* Edwards, 1929, and *Xenoplatyura*. Seven of these genera were analyzed in our dataset and group together with *Amerikeroplatus* and *Asiokeroplatus* (Figure 3). Within the *Cloeophoromyia*–*Asindulum* clade, the genus *Truplaya* appears to be paraphyletic with respect to *Xenoplatyura*, but this clade is supported only in the ML analysis (ufboot2 = 100). These two genera are also very similar morphologically, as noted e.g., by Matile [62] who also provided a simple phylogenetic tree in his paper.

Similarities of *Asiokeroplatus* and *Amerikeroplatus* were discussed by Fitzgerald [67]. They both have reduced mouthparts and palpi, and were, despite having unmodified antennae, placed in the tribe Keroplatini when described. The terminalia of *Asiokeroplatus tiger* Ševčík, Mantič and Blagoderov, 2015 is surprisingly similar to those of *Urytalpa* and agrees fully with the *Cloeophoromyia*–*Asindulum* group as defined above. The terminalia of *Amerikeroplatus dimorphicus* Fitzgerald, 2019 is insufficiently described and illustrated with respect to the presence/absence of gonocoxal apodemes and the outfit of the internal aedeagal complex. The description only notes that the latter is relatively small and “not anteriorly elongated into segment 7, at most slightly protruding into segment 8” [67]. *Pyratula* Edwards, 1929 forms a sister group to the *Cloeophoromyia*–*Asindulum* clade (Figure 3). It also has a small version of the aedeagal complex, with slightly elongated ejaculatory apodeme, but at the same time possess distinctly developed gonocoxal apodemes (see [68]).

The *Cloeophoromyia*–*Asindulum* group needs to be further revised, especially with respect to the possible inclusion of *Amerikeroplatus* and *Pyratula*. A separate tribal status may prove to more accurately reflect this lineage of the Keroplatinae, and thus, reduce the complexity of the Orfeliini and Keroplatini tribes as currently defined.

The position of *Platyura*, *Paleoplatyura*, *Burmacrocera* (including the related extant genus) and *Schizocyttara*, formerly considered as belonging to Orfeliini, is discussed in the previous sections. Another genus of uncertain position is *Chetoneura*, which was originally placed into the tribe Orfeliini but subsequently it was moved to Keroplatini, based on several morphological characters [3,58]. According to our data, the *Chetoneura* clade (ufboot2 = 100/PP = 1.00, including also *Laurypta leptura* (Edwards, 1928) is grouped with several Orfeliini genera (*Monocentrota Edwards*, 1925, *Platyceridion* Tollet, 1955 and *Proceroplatus* Edwards, 1925, ufboot2 = 98/PP = 0.99). Interestingly, the two latter genera with myrmecophagous larvae (*Platyceridion* and *Proceroplatus*) were found to be closely related, with maximum support (ufboot2 = 100/PP = 1). This clade forms a sister branch to *Monocentrota*, with high support (ufboot2 = 97/PP = 1), sharing with the sister genera several morphological features, e.g., setose laterotergite. The third genus of Orfeliini, reported by Kovac and Matile [69] as having myrmecophagous larvae, *Truplaya* Edwards, 1929, is not closely related (Figure 3), suggesting that this trophic strategy most probably evolved independently in at least two lineages of keroplatids.

The Keroplatini clade (with several problematic genera removed as mentioned above) represents a monophyletic group, although with relatively low support in ML (ufboot2 = 79/PP = 0.99), but with maximum support (ufboot2 = 100/PP = 1) without *Cerotelion* Rondani, 1856. The latter genus thus has an uncertain position and appears not closely related to *Rocetelion* Matile, 1988, although they were formerly (before Matile [64]) treated as a single genus and Matile [2] included both the genera into the subtribe Cerotelionina. Most other intergeneric relationships are in agreement with the phylogeny, based on morphology as proposed by Matile [2] (p. 532).

Species of the genera *Heteropterna* Skuse, 1888 and *Ctenoceridion* Matile, 1972 belong to a single well-supported (ufboot2 = 100/PP = 1) monophyletic clade, but not grouped together according to the present concept of the genera, indicating a possible generic synonymy (Figure 3). The presence or absence of pectinate antennae, the most dominant morphological difference between these two genera, is most probably a homoplastic character, not a synapomorphy [70,71]. On the other hand, the male terminalia of both genera are very similar, forming a natural group (see [2]). Accordingly, we propose the following synonymy at the genus level: *Heteropterna* Skuse, 1888 = *Ctenoceridion* Matile, 1972 syn. nov.

The genus *Platyroptilon* Westwood, 1850 may potentially be paraphyletic (Figure 3), but relationships within this clade are not well resolved. Three genera are included in the well-supported clade (ufboot2 = 100/PP = 1), *Platyroptilon*, *Setostylus* Matile, 1990 and *Xenokeroplatus* Matile, 1990; all of them are mostly tropical in distribution. This topology is in concordance with the phylogeny of Keroplatini proposed by Matile [2] (p. 532).

Several genera from our dataset remained as singletons, unplaced to a larger clade, e.g., the recently described Afrotropical genus *Kibaleana*, the mostly Australasian and Neotropical *Pyrtaula* Edwards, 1929, and, surprisingly, *Isoneuromyia* Brunetti, 1912. *Isoneuromyia* was initially included as a subgenus of *Orfelia*, together with some other subgenera, currently classified as genera. The close relationship of *Isoneuromyia* with *Orfelia* is not supported by present analysis (Figure 3), but rather a more ancient origin of *Isoneuromyia* could be inferred. This would be in agreement with Matile [2] who considered the shape of the male terminalia of *Isoneuromyia* as a primitive condition, due to its simple, macrocerine-looking gonostyli with bidentate apices.

The whole subfamily Keroplatinae is in need of generic revision since both its tribes, as currently defined morphologically, appear not to be monophyletic. Several characters used in the keys to delimitate genera, like mouthparts prolongation or reduction, or pectination of the antennae, have apparently evolved multiple times in the history of this family and these characters thus fail to define particular genera. On the other hand, the general morphology of the male terminalia, especially its internal structures, shows potential for being used to define morphological synapomorphies at higher taxonomic levels aligning with the molecular data presented here. This should be investigated further.

## 4. Conclusions

The family Keroplatidae consists of more than a thousand extant species in almost one hundred genera. It is a highly diversified and morphologically and ecologically disparate family, with the estimated origin in the Early Cretaceous period (145–100 mya). The whole group has historically been classified as a subfamily of the family Mycetophilidae, from which it has been later separated, together with other families of Sciaroidea. The sister family to Keroplatidae is still not clear, although there are some indications that it could be Bolitophilidae [30].

In this paper, we propose a new classification of the family Keroplatidae, with five subfamilies included: Arachnocampinae, Platyurinae stat. nov., Sciarokeroplatinae, Macrocerinae, Lygistorrhininae stat. nov., and Keroplatinae. We retain the current concept of the subfamily Macrocerinae (sensu Matile 1990 [2]) pending further molecular studies covering also other genera of Macrocerinae sensu lato, not included in this study. The traditional division of the subfamily Keroplatinae into the tribes Keroplatini and Orfeliini is not supported by the present analysis, and the emerging alternative tribal classification needs further work to be substantiated. It is suggested that reduced or prolonged mouthparts or pectinate antennae appeared repeatedly in the history of this family, and therefore, these characters should not be used as synapomorphies of particular genera nor higher taxa. Instead, the potential for using general morphology of male terminalia to define morphological synapomorphies at higher taxonomic levels should be investigated further.

The fact that new species and even genera are continually being described makes the phylogenetic analysis of the family difficult. In our dataset, almost half of the genera currently described in Keroplatidae are still missing. A more detailed study of larval stages represents another challenge, as they are still unknown in the vast majority of keroplatid taxa, and which may provide an interesting set of characters potentially useful for the reconstruction of the peculiar relationships in this family.

## Supplementary Materials

The following are available online at https://www.mdpi.com/2075-4450/11/6/348/s1, Table S1: List of specimens used for the phylogenetic analysis of Keroplatidae (Diptera) with GenBank accession numbers, Table S2: Primers for PCR amplification and sequencing of the mitochondrial and nuclear gene markers used in this study, Figure S1: Bayesian hypothesis for relationships among selected taxa of Keroplatidae based on DNA sequence data (12S, 16S, 18S, 28S, CAD, COI, cytB, and MCS).

## References

[1] Ševčík J., Kaspřák D., Mantič M., Fitzgerald S., Ševčíková T., Tóthová A., Jaschhof M. (2016). Molecular phylogeny of the megadiverse insect infraorder Bibionomorpha sensu lato (Diptera). PeerJ.

[2] Matile L. (1990). Recherches sur la systématique et lévolution des Keroplatidae: Diptera, Mycetophiloidea. Mem. Du Mus. Natl. D’histoire Nat. Ser. A Zool..

[3] Evenhuis N. (2006). Catalog of The Keroplatidae of The World (Insecta: Diptera).

[4] Ševčík J., Mantič M., Blagoderov V. (2015). Two new genera of Keroplatidae (Diptera), with an updated key to the World genera of Keroplatini. Acta Entomol. Musei Natl. Pragae.

[5] Kurina O., Mantič M., Ševčík J. (2017). A Remarkable New Genus of Keroplatidae (Insecta, Diptera) from the Afrotropical Region, with DNA Sequence Data. Afr. Invertebr..

[6] Fungus Gnats Online. http://sciaroidea.info/.

[7] Evenhuis N. (1994). Catalogue of The Fossil Flies of the World (Insecta: Diptera).

[8] Papp L., Ševčík J. (2005). Sciarokeroplatinae, a new subfamily of Keroplatidae (Diptera). Acta Zool. Acad. Sci. Hung..

[9] Blagoderov V., Grimaldi D. (2004). Fossil Sciaroidea (Diptera) In Cretaceous Ambers, Exclusive of Cecidomyiidae, Sciaridae, and Keroplatidae. Am. Mus. Novit..

[10] Grimaldi D., Amorim D.D.S., Blagoderov V. (2003). The Mesozoic Family Archizelmiridae (Diptera: Insecta). J. Paleontol..

[11] Baker C., Graham G., Scott K., Cameron S., Yeates D., Merritt D. (2008). Distribution and Phylogenetic Relationships of Australian Glow-Worms *Arachnocampa* (Diptera, Keroplatidae). Mol. Phylogenet. Evol..

[12] Saigusa T. (2018). Rare Sciaroid Genera from the Temperate east Palaearctic Region (Diptera: Sciaroidea). Jpn. J. Syst. Entomol..

[13] Bechev D. (2010). Flower visitation of fungus gnats from the genera *Antlemon*, *Asindulum* and *Macrorrhyncha* (Diptera: Keroplatidae): Published data and a new record. ZooNotes.

[14] Mantič M., Ševčík J. (2017). *Macrocera rohaceki* sp. nov. and other interesting records of Keroplatidae (Diptera) from southern and central Europe, with DNA sequence data. Acta Entomol. Musei Natl. Pragae.

[15] Reiter N., Freestone M., Brown G., Peakall R. (2019). Pollination by sexual deception of fungus gnats (Keroplatidae and Mycetophilidae) in two clades of Pterostylis (Orchidaceae). Bot. J. Linn. Soc..

[16] Matile L. (1997). Phylogeny and evolution of the larval diet in the Sciaroidea (Diptera, Bibionomorpha) since the Mesozoic. The Origin of Biodiversity in Insects: Phylogenetic Tests of Evolutionary Scenarios.

[17] Sharpe M.L., Dearden P.K., Gimenez G., Krause K.L. (2015). Comparative RNA seq analysis of the New Zealand glowworm *Arachnocampa luminosa* reveals bioluminescence-related genes. BMC Genom..

[18] Falaschi R., Amaral D., Santos I., Domingos A., Johnson G., Martins A., Viroomal I., Pompéia S., Mirza J., Oliveira A. (2019). *Neoceroplatus betaryiensis* nov. sp. (Diptera: Keroplatidae) is the first record of a bioluminescent fungus-gnat in South America. Sci. Rep..

[19] Aiello A., Jolivet P. (1996). Myrmecophily in Keroplatidae (Diptera: Sciaroidea). J. N. Y. Entomol. Soc..

[20] Chandler P.J., Matile L. (1998). A new species of *Platyceridion* Tollet (Diptera, Keroplatidae) with a larva predatory in ant infested internodes of *Humboldtia laurifolia* Vahl. Studia Dipterol..

[21] Chandler P.J., Pijnakker J. (2009). Tropical fungus gnats established in nurseries in The Netherlands (Diptera: Keroplatidae) and Mycetophilidae. Br. J. Entomol. Nat. Hist..

[22] Hickman V.V. (1965). On *Planarivora insignis* gen. *et* sp. n. (Diptera: Mycetophilidae), whose larval stages in land planarians are parasitic. Pap. Proc. R. Soc. Tasman..

[23] Bertone M. (2018). Field Observations of *Lygistorrhina sanctaecatharinae* Thompson (Diptera: Sciaroidea). Proc. Entomol. Soc. Wash..

[24] Edwards F. (1925). British Fungus-Gnats (Diptera, Mycetophilidae). With A Revised Generic Classification of the Family. Trans. R. Entomol. Soc. Lond..

[25] Tuomikoski R. (1966). Systematic position of *Lygistorrhina* Skuse (Diptera, Mycetophiloidea). Ann. Entomol. Fenn..

[26] Hennig W. (1973). Ordnung Diptera (Zweiflügler). Handb. Der Zool..

[27] Søli G.E.E., Vokeroth J.R., Matile L., Papp L., Darvas B. (2000). Families of Sciaroidea. Contributions to A Manual of Palaeartic Diptera.

[28] Amorim D.D.S., Rindal E. (2007). Phylogeny of the Mycetophiliformia, with proposal of the subfamilies Heterotrichinae, Ohakuneinae, and Chiletrichinae for the Rangomaramidae (Diptera, Bibionomorpha). Zootaxa.

[29] Hippa H., Vilkamaa P. (2006). Phylogeny of the Sciaroidea (Diptera): The Implication of additional taxa and character data. Zootaxa.

[30] Wiegmann B.M., Trautwein M.D., Winkler I.S., Barr N.B., Kim J.-W., Lambkin C., Bertone M.A., Cassel B.K., Bayless K.M., Heimberg A.M. (2011). Episodic radiations in the fly tree of life. Proc. Natl. Acad. Sci. USA.

[31] Vilkamaa P., Hippa H. (2005). The genus Sciarotricha gen. n. (Sciaridae) and the phylogeny of recent and fossil Sciaroidea (Diptera). Insect Syst. Evol..

[32] Bertone M., Courtney G., Wiegmann B. (2008). Phylogenetics and temporal diversification of the earliest true flies (Insecta: Diptera) based on multiple nuclear genes. Syst. Entomol..

[33] Rindal E., Søli G.E., Bachmann L. (2009). Molecular phylogeny of the fungus gnat family Mycetophilidae (Diptera, Mycetophiliformia). Syst. Entomol..

[34] Kallweit U. *Lygistorrhina* Skuse has branched from the Keroplatidae (Diptera: Sciaroidea)—A new perspective on the phylogenetic relationships in the keroplatid group of fungus gnats. Proceedings of the 8th International Congress of Dipterology.

[35] Thompson F.C. (1975). Notes on the genus *Lygistorrhina* Skuse with the description of the first Nearctic species (Diptera: Mycetophiloidea). Proc. Entomol. Soc. Wash..

[36] Kaspřák D., Kerr P., Sýkora V., Tóthová A., Ševčík J. (2019). Molecular phylogeny of the fungus gnat subfamilies Gnoristinae and Mycomyinae, and their position within Mycetophilidae (Diptera). Syst. Entomol..

[37] Sikora T., Jaschhof M., Mantič M., Kaspřák D., Ševčík J. (2019). Considerable congruence, enlightening conflict: Molecular analysis largely supports morphology-based hypotheses on Cecidomyiidae (Diptera) phylogeny. Zool. J. Linn. Soc..

[38] Katoh K., Standley D.M. (2013). MAFFT Multiple Sequence Alignment Software Version 7: Improvements in Performance and Usability. Mol. Biol. Evol..

[39] Castresana J. (2000). Selection of conserved blocks from multiple alignments for their use in phylogenetic analysis. Mol. Biol. Evol..

[40] Xia X., Lemey P. (2009). Assessing substitution saturation with DAMBE. Phylogenet. Handb. Pract. Approach DNA Protein Phylogeny.

[41] Xia X. (2013). DAMBE5: A comprehensive software package for data analysis in molecular biology and evolution. Mol. Biol. Evol..

[42] Lanfear R., Frandsen P.B., Wright A.M., Senfeld T., Calcott B. (2016). PartitionFinder 2: New methods for selecting partitioned models of evolution for molecular and morphological phylogenetic analyses. Mol. Biol. Evol..

[43] Yang Z. (1996). Among-site rate variation and its impact on phylogenetic analyses. Trends Ecol. Evol..

[44] Minh B.Q., Schmidt H.A., Chernomor O., Schrempf D., Woodhams M.D., Von Haeseler A., Lanfear R. (2020). IQ-TREE 2: New models and efficient methods for phylogenetic inference in the genomic era. Mol. Biol. Evol..

[45] Hoang D.T., Chernomor O., Haeseler A.V., Minh B.Q., Vinh L.S. (2017). UFBoot2: Improving the Ultrafast Bootstrap Approximation. Mol. Biol. Evol..

[46] Ronquist F., Huelsenbeck J.P. (2003). MRBAYES 3: Bayesian phylogenetic inference under mixed models. Bioinformatics.

[47] Miller M.A., Pfeiffer W., Schwartz T. Creating the CIPRES Science Gateway for inference of large phylogenetic trees. Proceedings of the 2010 gateway computing environments workshop (GCE).

[48] Rambaut A., Drummond A.J., Xie D., Baele G., Suchard M.A. (2018). Posterior summarization in Bayesian phylogenetics using Tracer 1.7. Syst. Biol..

[49] Letunic I., Bork P. (2011). Interactive Tree Of Life v2: Online annotation and display of phylogenetic trees made easy. Nucleic Acids Res..

[50] Edwards F.W. (1924). A note on the “New Zealand glow-worm” (Diptera, Mycetophilidae). Ann. Mag. Nat. Hist..

[51] Sabrosky C.W. (1999). Family-group names in Diptera: An annotated catalog. Myia.

[52] Soós Á., Papp L. (1988). Catalogue of Palaearctic Diptera: Ceratopogonidae-Mycetophilidae.

[53] Kurina O. (1998). Fungus Gnats in Estonia: (Diptera: Bolitophilidae, Keroplatidae, Macroceridae, Ditomyiidae, Diadocidiidae, Mycetophilidae).

[54] Polevoi A.V. (2000). Fungus Gnats (Diptera: Bolitophilidae, Ditomyiidae, Keroplatidae, Diadocidiidae, Mycetophilidae) of Karelia.

[55] Papp L. (2006). New record of Diptera species from Hungary, with the list of the Hungarian Scathophagidae. Folia Entomol. Hung..

[56] Papp L. (2007). Dixidae, Axymyiidae, Mycetobiidae, Keroplatidae, Macroceridae, and Ditomyiidae(Diptera) from Taiwan. Acta Zool. Acad. Sci. Hung..

[57] Ševčík J. (2009). *Langkawiana maculata* gen. et sp. n. from Malaysia and its systematic position in the tribe Robsonomyiini (Diptera: Keroplatidae). Zootaxa.

[58] Amorim D.D.S., Niu C., Li X., Lei C., Clarke A. (2008). *Chetoneura shennonggongensis*, a new species of cave-dwelling Keroplatini from China (Diptera: Keroplatidae), with a discussion of the position of *Chetoneura*. Zootaxa.

[59] Blagoderov V., Kirk–Spriggs A.H., Sinclair B.J. (2017). Lygistorrhinidae (Long-beaked Fungus Gnats). Manual of Afrotropical Diptera. Nematocerous Diptera and lower Brachycera.

[60] Hippa H., Mattsson I., Vilkamaa P. (2005). New Taxa of the Lygistorrhinidae (Diptera: Sciaroidea) and Their Implications for a Phylogenetic Analysis of the Family. Zootaxa.

[61] Blagoderov V., Hippa H., Ševčík J. (2009). *Asiorrhina*, a new Oriental genus of Lygistorrhinidae (Diptera: Sciaroidea) and its phylogenetic position. Zootaxa.

[62] Matile L. (1978). Revision des *Truplaya* Afrotropicaux (Diptera, Mycetophilidae). Ann. De La Société Entomol. De Fr..

[63] Blagoderov V., Ševčík J., Kirk–Spriggs A.H., Sinclair B.J. (2017). Keroplatidae (Predaceous Fungus Gnats). Manual of Afrotropical Diptera. Nematocerous Diptera and lower Brachycera.

[64] Matile L. (1988). *Rocetelion*, a new Holarctic genus of the Keroplatidae (Diptera, Mycetophiloidea): Description, phylogenetic and biogeographic notes. Ann. Entomol. Fenn..

[65] Uesugi K. (2004). Fungus gnats of the genus Urytalpa Edwards (Diptera: Keroplatidae) in Japan. Entomol. Sci..

[66] Kjærandsen J., Martinsson S., Hedmark K., Evenhuis N.L. (2009). On the genus *Urytalpa* Edwards (Diptera: Keroplatidae) in the Nordic and Nearctic Regions, with fixation of a new type species and a key to World males. Zootaxa.

[67] Fitzgerald S. (2019). A Curious new genus of Keroplatini (Diptera: Keroplatidae) from Guatemala. Zootaxa.

[68] Chandler P.J., Blasco-Zumeta J. (2001). The fungus gnats (Diptera, Bolitophilidae, Keroplatidae and Mycetophilidae) of the Monegros region (Zaragoza, Spain) and five other new European species of *Pyratula* Edwards and *Sciophila* Meigen. Zapateri. Rev. Aragonesa De Entomol..

[69] Kovac D., Matile L. (1997). *Truplaya ferox* (Insecta: Diptera: Mycetophiloidea), a new Malaysian keroplatid from bamboo phytotelmata with larvae predaceous on ants. Raffles Bull. Zool..

[70] Matile L. (1974). Diptera: Mycetophilidae Keroplatinae. S. Afr. Anim. Life.

[71] Ševčík J. (2012). *Terocelion* gen. nov., a new Oriental genus of Keroplatidae (Diptera) with pectinate antennae. Acta Entomol. Musei Natl. Pragae.

