# Supplementary material for: Hidden in Plain Sight: Comprehensive Molecular Phylogeny of Keroplatidae and Lygistorrhinidae (Diptera) Reveals Parallel Evolution and Leads to a Revised Family Classification
Source: Insects. 2020 Jun 4;11(6):348. doi: 10.3390/insects11060348 (PMC7349917; doi:10.3390/insects11060348)

Table S1: List of specimens used for the phylogenetic analysis of Keroplatidae (Diptera) with GenBank accession numbers

Species	Voucher	12S	16S	18S	28S	CAD	COI	cytB	MCS	Geographic origin
Afrokeroplatus ashleyi Ševčík, Mantič, Blagoderov, 2015	K44	MT446516	MT446593	MT446732	MT446823	n/a	MT446917	n/a	MT535446	Republic of South Africa
Amerikeroplatus dimorphicus Fitzgerald, 2019	K107	n/a	MT446623	MT446762	MT446857	n/a	MT446946	MT447026	MT535437	Guatemala
Angazidzia insolita (Matile, 1972)	K32	MT446511	n/a	MT446727	MT446817	n/a	n/a	n/a	n/a	Republic of South Africa
Antlemon (*A.*) *halidayi* (Loew, 1871)	K5	MT446489	MT446566	MT446705	MT446794	MT446642	MT446892	MT446974	MT535457	Spain (Mallorca)
Antlemon (*Antlemonopsis*) *brevimanum* (Loew, 1871)	K6	MT446490	MT446567	MT446706	MT446795	MT446643	MT446893	MT446975	MT535458	Slovakia
Arachnocampa (*A.* s. str.) *luminosa* (Skuse, 1891)	aralum		MT446557	MT446691	MT446775	MT446633	MT446877	MT446959	MT535401	New Zealand
	K104a	MT446546								New Zealand
Arachnocampa (*C.*) *flava* Harrison, 1966	arafla	NC_016204	NC_016204	KC177277	KC177644	KC_178393	NC_016204	NC_016204	n/a	Australia
Asindulum montanum Roder, 1887	K47	MT446517	MT446594	MT446733	MT446824	MT446660	MT446918	MT447000	MT535466	USA
Asiokeroplatus tiger Ševčík, Mantič, Blagoderov, 2015	K34	MT446512	MT446588	n/a	MT446818	n/a	n/a	n/a	n/a	Thailand
Asiorrhina parasiatica Blagoderov, Hippa, Ševčík, 2009	B4	KP288675	KP288709	KP288744	MT446776	KX453715	KT316832	MT446960	MT535455	Thailand
Bibio marci (Linnaeus, 1758)	bibmar						KT316846			Unknown (from GenBank)
	OUT1	KJ136689	KJ136724	KP288758	MT446865	KX453730		GATJ00000000.2	MH114581	Czech Republic
Blagorrhina sp. (undescribed species)	OUT20	KP288694	KP288726	KP288772	MT446868	KX453739	KT316862	MT447034	MT535454	Malaysia
Bolitophila cinerea Meigen, 1818	bolcin			MT446695	MT446780	MT446636	MT446881	MT446964	MH114583	Czech Republic
	B1	KJ136677	KJ136712							Slovakia
Bolitophila hybrida (Meigen, 1804)	bolhyb	MT446482	MT446560	MT446696	MT446781	MT446637		MT446965	MT535471	Czech Republic
	BN3						MT446882			Czech Republic
Catotricha subobsoleta (Alexander, 1924)	catsub			MT446697	MT446782		MT446883	MH114511	MH114582	USA
	OUT42	KP288706	MG554124			KX453747				USA
Cerotelion racovitzai Matile & Burghele-Balacesco, 1969	K2	MT446486	MT446563	MT446702	MT446791	MT446640	MT446889	MT446971	MT535443	Slovakia
Cerotelion striatum (Gmelin, 1790)	K2b	MT446487	MT446564	MT446703	MT446792	n/a	MT446890	MT446972	MT535444	Czech Republic
Chetoneura cavernae Colless, 1962	K9	MT446494	MT446571	MT446710	MT446799	n/a	MT446897	MT446979	MT535414	Brunei
Chetoneura sp. (undescribed species)	K59	MT446523	MT446599	n/a	MT446831	n/a	MT446924	n/a	MT535447	Brunei
Chiasmoneura anthracina Meijere, 1913	B6	KJ136682	KJ136717	KP288745	MT446777	n/a	KT316833	MT446961	MT535442	Thailand
Ctenoceridion freemani Matile, 1972	K39b	MT446514	MT446590	MT446729	MT446820	MT446657	MT446915	MT446998	MT535423	Madagascar
Ctenoceridion sp. (undescribed species)	K56	MT446522	MT446598	MT446738	MT446830	MT446663	MT446923	MT447004	MT535429	Madagascar
Diadocidia ferruginosa (Meigen, 1830)	diafer				MT446785		MT446885	MT446968		Czech Republic
	SJ1	KC435526	KC435562	KP288786		KX453752			MH114576	Czech Republic
Diadocidia globosa Papp & Ševčík, 2005	SJ9c	KP288708	KP288740	KP288789	MT446874	KX453755	KT316878	MT447039	MT535470	Thailand
Ditomyia fasciata (Meigen, 1818)	ditfas			MT446699	MT446786		MT446886		MH114585	Czech Republic
	SJ3	KJ136698	KJ136734			KX453753		MH114518		Czech Republic
Euceroplatus sp. 1 (undescribed species)	BA24	MT446481	MT446558	MT446693	MT446778	MT446635	MT446879	MT446963	MT535411	Thailand
Euceroplatus sp. 2 (undescribed species)	K26	MT446506	MT446583	MT446722	MT446812	MT446653	MT446909	MT446992	MT535421	Brunei
genus near *Burmacrocera* sp. 1 (undescribed species)	K8	MT446492	MT446569	MT446708	MT446797	MT446645	MT446895	MT446977	MT535459	Brunei
genus near *Burmacrocera* sp. 2 (undescribed species)	K8e	MT446493	MT446570	MT446709	MT446798	MT446646	MT446896	MT446978	MT535463	Taiwan
genus near *Robsonomyia* (undescribed species)	K106	n/a	n/a	MT446761	MT446856	n/a	n/a	n/a	MT535452	USA
Gnoriste bilineata Zetterstedt, 1852	GS4	KP288679	KP288713	KP288752	MT446787	KX453720	KT316839	KT923597	MH114535	Czech Republic
Heteropeza pygmaea Winnertz, 1846	hepyg	MG684450	MT446561	MT446700	MT446788	MT446638	MT446887	MT446969	MT535402	Slovakia
Heteropterna perdistincta Matile, 1990	K66	MT446528	MT446604	MT446743	MT446837	MT446667	n/a	MT447009	MT535431	French Guyana
Heteropterna sp. (undescribed species)	K55	MT446521	MT446597	MT446737	MT446829	n/a	MT446922	MT447003	MT535428	Madagascar
Heterotricha takkae Chandler, 2002	hettak	MG684499	MG684543	MH114228	MT446789	MH114336	MH114452	MH114510	MH114584	Greece
Isoneuromyia pseudochracea (Landrock, 1925)	K41	MG049746	MT446591	MT446730	MT446821	MT446658	MG049753	MG049759	MT535424	Slovakia
Isoneuromyia semirufa (Meigen, 1818)	K84	MG049748	MT446615	MT446754	MT446849	MT446676	MG049754	MG049761	n/a	Czech Republic
Keroplatus testaceus (Dalman, 1818)	kertes			MT446763	MT446858	MT446680	MT446947	MT447027	MT535403	Czech Republic
	B7	KJ136683	KJ136718							Slovakia

Keroplatus tipuloides (Bosc, 1792)	K1	MT446485	MT446562	MT446701	MT446790	MT446639	MT446888	MT446970	MT535412	Slovakia
Kibaleana apicospinosa Kurina, Mantič & Ševčík, 2017	K60	MT446524	MT446600	MT446739	MT446832	n/a	MT446925	MT447005	MT535430	Uganda
Laurypta leptura (Edwards, 1928)	K14	MT446497	MT446574	MT446713	MT446803	MT446649	MT446900	MT446983	MT535416	Brunei
Lestremia cinerea Macquart, 1826	lescin				MT446859	n/a			MT535404	Slovakia
	OUT6	KJ136693	KJ136728	KP288761			KT316850	MT447032		Slovakia
Lygistorrhina (*L.* s. str.) sp. (undescribed species)	K70	MT446532	MT446608	MT446747	MT446841	MT446670	MT446932	MT447013	MT535449	Brunei
Lygistorrhina (*P.*) *cerqueirai* Lane, 1958	K61	KX453690	KX453697	KX453703	MT446833	KX453724	KX453759	MH114487	MH114565	French Guyana
Lygistorrhina (*P.*) *sanctaecatherinae* Thompson, 1975	lygsan		MT446624	MT446764	MT446860	MT446681	MT446948	MT447028	MT535405	USA
	K105	MT446547								USA
Lyprauta sp. (undescribed species)	K64	MT446526	MT446602	MT446741	MT446835	MT446665	MT446927	MT447007	MT535467	French Guyana
Macrocera centralis Meigen, 1818	K10	KP288682	KP288716	KP288755	MT446800	KX453723	KT316841	MT446980	MT535464	Slovakia
Macrocera vittata Meigen, 1830	macvit	MT446548	MT446625	MT446765	MT446861	MT446682	MT446949	MT447029	MT535453	Czech Republic
Macrorrhyncha collarti (Tollet, 1955)	K73	MT446533	MT446609	MT446748	MT446842	MT446671	MT446933	MT447014	n/a	Slovakia
Macrorrhyncha flava Winnertz, 1846	K7	MT446491	MT446568	MT446707	MT446796	MT446644	MT446894	MT446976	MT535462	Slovakia
Manota unifurcata Lundstrom, 1913	manuni			MT446766	MT446862	MT446683	MT446950	MT447030	n/a	Czech Republic
	S8	KC435542	KC435578							Slovakia
Matileola sp. (undescribed species)	K97	MT446543	MT446620	MT446758	MT446853	MT446678	MT446943	MT447023	n/a	Taiwan
Micrapemon sp. (undescribed species)	K15b	MT446498	MT446575	MT446714	MT446804	n/a	MT446901	MT446984	MT535417	French Guyana
Microkeroplatus minutus Ševčík & Papp, 2011	K16	MT446499	MT446576	MT446715	MT446805	n/a	MT446902	MT446985	MT535418	Brunei
Monocentrota lundstroemi Edwards, 1925	K11	MT446495	MT446572	MT446711	MT446801	MT446647	MT446898	MT446981	MT535415	Slovakia
Monocentrota matilei Bechev, 1989	K69	MT446531	MT446607	MT446746	MT446840	MT446669	MT446931	MT447012	MT535448	Slovakia
Neoceroplatus sp. (undescribed species)	K62	MT446525	MT446601	MT446740	MT446834	MT446664	MT446926	MT447006	n/a	French Guyana
Neoplatyura nigricauda (Strobl, 1893)	K12	MT446496	MT446573	MT446712	MT446802	MT446648	MT446899	MT446982	n/a	Slovakia
Neoplatyura noorae Salmela, 2014	K77	MT446536	MT446612	MT446751	MT446845	MT446674	MT446936	MT447017	MT535433	Finland
Nepaletricha furcata Hippa, Chandler & Papp, 2009	OUT10c	KP288685	KP288718	KP288764	n/a	KX453733			n/a	Thailand
	OUT10e						MT446953	MT447033		Thailand
Nepaletricha sp. (undescribed species)	nepsp	MT446549				n/a		MT447031		Thailand
	BA25a		MT446559	MT446694	MT446779		MT446880		MT535460	Thailand
Nervijuncta sp. (undescribed species)	D11	MT446484	n/a	MT446698	MT446784	n/a	MT446884	MT446967	n/a	New Zealand
Orfelia fasciata (Meigen, 1804)	K37	MT446513	MT446589	MT446728	MT446819	MT446656	MT446914	MT446997	n/a	Slovakia
Orfelia nemoralis (Meigen, 1818)	orfnem			MT446768	MT446864	n/a	MT446952	n/a	MT535406	Slovakia
	K3	KP288681	KP288715							Czech Republic
Paleoplatyura johnsoni Johannsen, 1910	pajo	MT446551	MT446627	MT446769			MT446954	MT447035	MT535407	Italy
	K80				MT446846	MT446675				Italy
Paleoplatyura melanderi Fisher, 1941	K51	MT446520	MT446596	MT446736	MT446827	MT446662	MT446921	n/a	MT535427	USA
Placoceratias sp. (undescribed species)	K27	MT446507	MT446584	MT446723	MT446813	MT446654	MT446910	MT446993	n/a	Peru
Platyceridion sp. (undescribed species)	K81a	MT446537	MT446613	MT446752	MT446847	n/a	MT446937	MT447018	MT535434	China
Platyroptilon sp. 1 (undescribed species)	K18	MT446500	MT446577	MT446716	MT446806	MT446650	MT446903	MT446986	MT535419	Thailand
Platyroptilon sp. 2 (undescribed species)	K67	MT446529	MT446605	MT446744	MT446838	n/a	MT446929	MT447010	MT535469	French Guyana
Platyura marginata Meigen, 1804	K4	MT446488	MT446565	MT446704	MT446793	MT446641	MT446891	MT446973	MT535413	Slovakia
Platyura pectoralis Coquillett, 1895	K48	MT446518	MT446595	MT446734	MT446825	MT446661	MT446919	MT447001	MT535426	USA
Porricondyla nigripennis (Meigen, 1830)	OUT16	KP288690	KP288722	KP288768	MT446867	KX453736	KT316859	n/a	n/a	Slovakia
Proceroplatus sp. 1 (undescribed species)	K25	MT446505	MT446582	MT446721	MT446811	n/a	MT446908	MT446991	n/a	Brunei
Proceroplatus sp. 2 (undescribed species)	K83	MT446538	MT446614	MT446753	MT446848	n/a	MT446938	MT447019	n/a	China
Pyratula subcanariae Chandler & Blasco-Zumeta, 2001	K74	MT446534	MT446610	MT446749	MT446843	MT446672	MT446934	MT447015	MT535450	Finland
Pyratula zonata (Zetterstedt, 1855)	K30	MT446509	MT446586	MT446725	MT446815	MT446655	MT446912	MT446995	MT535445	Czech Republic
Pyrtaula sp. (undescribed species)	K92	MT446540	MT446617	MT446756	MT446851	MT446677	MT446940	MT447020	MT535436	New Zealand
Ralytupa pendleburyi (Edwards, 1928)	K19	MT446501	MT446578	MT446717	MT446807	n/a	MT446904	MT446987	n/a	Brunei
Ralytupa sp. (undescribed species)	K31	MT446510	MT446587	MT446726	MT446816	n/a	MT446913	MT446996	n/a	Democratic Republic of Congo
genus near *Robsonomyia* sp. (undescribed species)	K53	KP288683	n/a	KP288756	MT446828	n/a	KT316842	n/a	n/a	USA

Rocetelion humerale (Zetterstedt, 1850)	K68	MT446530	MT446606	MT446745	MT446839	MT446668	MT446930	MT447011	MT535432	Slovakia
Rutylapa ruficornis (Zetterstedt, 1851)	B8	KJ136684	KJ136719	MT446692	n/a	MT446634	MT446878	MT446962	n/a	Turkey
Schizocyttara turneri Matile, 1974	K43	MT446515	MT446592	MT446731	MT446822	MT446659	MT446916	MT446999	MT535425	South Africa
Schwenckfeldina carbonaria (Meigen, 1830)	SCI12	MT446552	MG554120	MG554133	MT446870	MT446686	MG554164	MT447036	MT535439	Slovakia
Sciara hemerobioides (Scopoli, 1763)	scihem	MT446553	MT446628	MT446770	MT446871	MT446687	MT446955	MT447037	MT535408	Slovakia
Sciarokeroplatus pileatus Papp & Ševčík, 2005	scipil			MT446771	MT446872	MT446688			MT535409	Taiwan
	K96	MT446542	MT446619				MT446942	MT447022		Taiwan
Sciarosoma nigriclava (Strobl, 1898)	OUT13b	KP288688	KP288720	KP288766	MT446866	MT446685	KT316857	n/a	MT535438	Finland
Sciophila rufa Meigen, 1830	sciruf	MT446554	MT446629	MT446772	MT446873	MT446689	MT446956	MT447038	MT535410	Czech Republic
Setostylus sp. 1 (undescribed species)	K20c	MT446502	MT446579	MT446718	MT446808	MT446651	MT446905	MT446988	MT535420	Brunei
Setostylus sp. 2 (undescribed species)	TW36	MT446556	MT446632	MT446774	MT446876	n/a	MT446958	MT447041	MT535441	Taiwan
Symmerus annulatus (Meigen, 1830)	D2	MT446483	KX453696	FJ171934	MT446783	KC177112	KX453757	MT446966	MT535461	Slovakia
Taulyrpa sp. (undescribed species)	K65	MT446527	MT446603	MT446742	MT446836	MT446666	MT446928	MT447008	MT535468	French Guyana
Taxicnemis hirta (Marshall, 1896)	NZ1	MT446550	MT446626	MT446767	MT446863	MT446684	MT446951	n/a	MT535456	New Zealand
Tergostylus sp. (undescribed species)	K90	MT446539	MT446616	MT446755	MT446850	n/a	MT446939	n/a	MT535435	Madagascar
Tolletia vrydaghi (Tollet, 1955)	K94	MT446541	MT446618	MT446757	MT446852	n/a	MT446941	MT447021	n/a	Democratic Republic of Congo
Truplaya (*Truplaya*) sp. 1 (undescribed species)	K98	MT446544	MT446621	MT446759	MT446854	MT446679	MT446944	MT447024	MT535451	Brunei
Truplaya (*Truplayella*) sp. 2 (undescribed species)	K101	MT446545	MT446622	MT446760	MT446855	n/a	MT446945	MT447025	n/a	Cameroon
Urytalpa macrocera (Edwards, 1913)	K49	MT446519	MT472694	MT446735	MT446826	n/a	MT446920	MT447002	n/a	Czech Republic
Urytalpa trivittata (Lundstrom, 1914)	K76	MT446535	MT446611	MT446750	MT446844	MT446673	MT446935	MT447016	n/a	Finland
Xenokeroplatus sp. 1 (undescribed species)	K21	MT446503	MT446580	MT446719	MT446809	n/a	MT446906	MT446989	MT535465	Thailand
Xenokeroplatus sp. 2 (undescribed species)	TW33	MT446555	MT446630	MT446773	MT446875	MT446690	MT446957	MT447040	MT535440	Taiwan
Xenoplatyura sp. 1 (undescribed species)	K22	MT446504	MT446581	MT446720	MT446810	MT446652	MT446907	MT446990	n/a	Thailand
Xenoplatyura sp. 2 (undescribed species)	K28	MT446508	MT446585	MT446724	MT446814	n/a	MT446911	MT446994	MT535422	Brunei
Zygoneura sciarina Meigen, 1830	OUT33	KP288700	KP288732	KP288777	MT446869	KX453742	KT316867	MH114507	MH114573	Slovakia

Table S2 – Primers for PCR amplification and sequencing of the mitochondrial and nuclear gene markers used in this study

Gene region	Primer name	Direct ion	Primer sequences (5'→3')	Source
12S	12Sma	F	CTGGGATTAGATACCCTGTTAT	[1]
	12Smb	R	CAGAGAGTGACGGGCGATTTGT	
	SR-J-14199	F	TACTATGTTACGACTTAT	[2]
	MZ-12S-R	R	GCCAGCATTTGCGGTTATAC	Žurovcová lab
16S	dipt-16S-F	F	TAATCCAACATCGAGGTC	[3]
	dipt-16S-R	R	CGAAGGTAGCATAATCAGTAG	
18S	18SF	F	AACCTGGTTGATCCTGCCAGT	[4]
	18S-3-seq	R	GGTTAGAACTAGGGCGGTATCT	[5]
28S	dipt-28S-F	F	AGAGAGAGAGTTCAAGAGTACGTG	[6]
	dipt-28S-R	R	TAGTTCACCATCTTTCGGGTC	[7]
	28S-A2	F	GCTTAGGACCGACTAACTCG	Ševčík lab
	28S-R2	R	GGTTACGGAATTGGAACCG	
CAD	CAD-1057-F	F	ACNGAYTAYGAYATGTGYGA	[8]
	CAD-1278-R	R	TCRTTNTTYTTWGCRATYAAYTGCAT	
COI	LCO1490	F	GGTCAACAAATCATAAAGATATTGG	[9]
	HCO2198	R	TAAACTTCAGGGTGACCAAAAAATCA	
cytB	dipt-cytB-F	F	TATGTTTTATGAGGACAAATATC	[10]
	mycet_cytB-R	R	ATTACTCCCCCTAATTTATTAGGAAT	[11]
MCS	mMCS-1715	F	AAATATGATCGNGARTGGATGA	
	MCS1-R2b	R	TGGTCAATRCAAATCATTTGACA	

1. Koufopanou, V.; Reid, D. G.; Ridgway, S. A; Thomas, R. H. A molecular phylogeny of the Patellid limpets (Gastropoda: Patellidae) and its implications for the origins of their antitropical distribution. *Molecular Phylogenetics and Evolution* **1999**, *11*, 138–156.
2. Kambhampati, S; Smith, P. T. PCR primers for the amplification of four insect mitochondrial gene fragments. *Insect Molecular Biology* **1995**, *4*, 233–236.
3. Roháček, J.; Tothova, A.; Vaňhara, J. Phylogeny and affiliation of European Anthomyzidae (Diptera) based on mitochondrial 12S and 16S rRNA. *Zootaxa* **2009**, *2054*, 49-58.
4. Katana, A.; Kwiatowski, J.; Spalik, K.; Zakryś, B.; Szalacha, E; Szymańska, H. Phylogenetic position of *Koliella* (Chlorophyta) as inferred from nuclear and chloroplast small subunit rDNA. *Journal of* Phycology **2001**, *37*, 443–451.
5. Campbell, B. C.; Ross, G. F; Woodward, T. E. Paraphyly of Homoptera and Auchenorrhyncha inferred from 18S rDNA nucleotide sequence. *Systematic Entomology* **1995**, *20*, 175–194.

Table S2 – Primers for PCR amplification and sequencing of the mitochondrial and nuclear gene markers used in this study

6. Belshaw, R; Quicke, D. L. J. A molecular phylogeny of the Aphidiinae (Hymenoptera: Braconidae). *Molecular Phylogenetics and* Evolution **1997**, 7, 28–293.
7. Laurenne, N. M.; Broad, G. R; Quicke, D. L. J. Direct optimization and multiple alignment of 28S D2–D3 rDNA sequences: problems with indels on the way to a molecular phylogeny of the cryptine ichneumon wasps (Insecta: Hymenoptera), *Cladistics* **2006**, *22*, 442–473.
8. Moulton, J. K.; Wiegmann, B. M. Evolution and phylogenetic utility of CAD (rudimentary) among Mesozoic-aged Eremoneuran Diptera (Insecta). *Molecular phylogenetics and evolution* **2004**, *31*), 363-378.
9. Folmer, O.; Black, M.; Hoen, W.; Lutz, W; Vrijenhoek, R. DNA primers for amplification of mitochondrial cytochrome c oxidase subunit I from diverse metazoan 9 invertebrates. *Molecular Marine Biology and Biotechnology* **1994**, *3*, 294–299.
10. Su, K. F. I.; Kutty, S. N.; Meier, R. Morphology versus molecules: the phylogenetic relationships of Sepsidae (Diptera: Cyclorrhapha) based on morphology and DNA sequences data from ten genes. *Cladistics* **2008**, *24*, 902–916.
11. Kaspřák, D.; Kerr, P.; Sýkora, V.; Tóthová, A.; & Ševčík, J. Molecular phylogeny of the fungus gnat subfamilies Gnoristinae and Mycomyinae, and their position within Mycetophilidae (Diptera). *Systematic entomology* **2019**, *44*, 128-138.

Mantič *et al*. (2020): Hidden in plain sight: Comprehensive molecular phylogeny of Keroplatidae and Lygistorrhinidae (Diptera) reveals parallel evolution and leads to a revised family classification

Figure S1: Bayesian hypothesis for relationships among selected taxa of Keroplatidae based on DNA sequence data (12S, 16S, 18S, 28S, CAD, COI, cytB, and MCS).

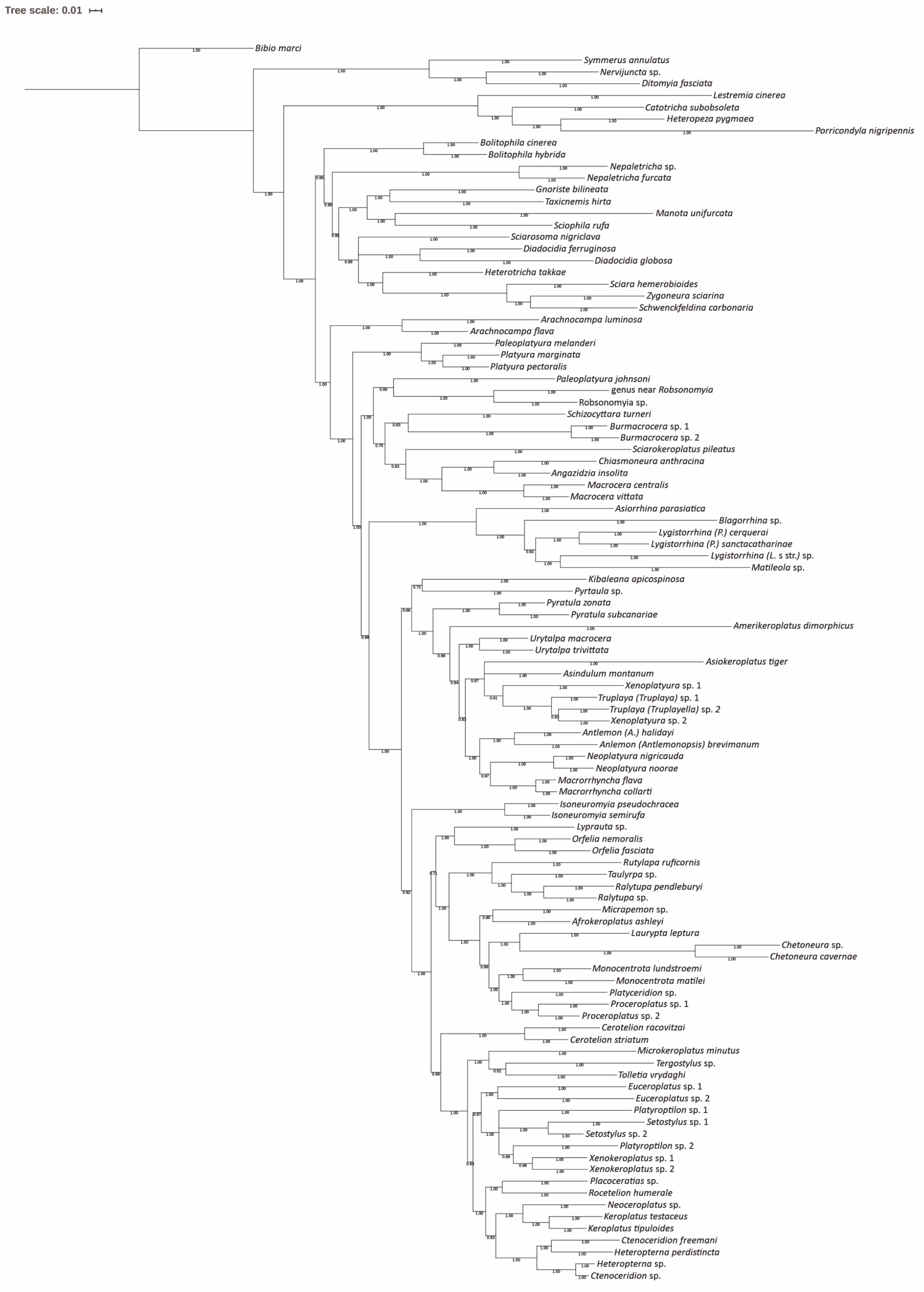

Supplement: Supplementary file 1 [file insects-11-00348-s001.pdf]
